# Solution Structure of SpoIVB Reveals Mechanism of PDZ Domain-Regulated Protease Activity

**DOI:** 10.3389/fmicb.2019.01232

**Published:** 2019-06-12

**Authors:** Xie Xie, Nannan Guo, Guangpu Xue, Daoqing Xie, Cai Yuan, Joshua Harrison, Jinyu Li, Longguang Jiang, Mingdong Huang

**Affiliations:** ^1^College of Chemistry, Fuzhou University, Fuzhou, China; ^2^College of Biological Science and Engineering, Fuzhou University, Fuzhou, China; ^3^Beth Israel Deaconess Medical Center, Harvard Medical School, Boston, MA, United States

**Keywords:** sporulation, regulated intramembrane proteolysis, SpoIVB, PDZ-protease, SAXS

## Abstract

Intramembrane proteases hydrolyze peptide bonds within the cell membrane as the decision-making step of various signaling pathways. Sporulation factor IV B protease (SpoIVB) and C-terminal processing proteases B (CtpB) play central roles in cellular differentiation via regulated intramembrane proteolysis (RIP) process which activates pro-σ^K^ processing at the σ^K^ checkpoint during spore formation. SpoIVB joins CtpB in belonging to the widespread family of PDZ-proteases, but much remains unclear about the molecular mechanisms and structure of SpoIVB. In this study, we expressed inactive SpoIVB (SpoIVB^S378A^) fused with maltose binding protein (MBP)-tag and obtained the solution structure of SpoIVB^S378A^ from its small angle X-ray scattering (SAXS) data. The fusion protein is more soluble, stable, and yields higher expression compared to SpoIVB without the tag. MBP-tag not only facilitates modeling of the structure in the SAXS envelope but also evaluates reliability of the model. The solution structure of SpoIVB^S378A^ fits closely with the experimental scattering data (χ^2^= 1.76). Comparing the conformations of PDZ-proteases indicates that SpoIVB adopts a PDZ-protease pattern similar to the high temperature requirement A proteases (HtrAs) rather than CtpB. We not only propose that SpoIVB uses a more direct and simple way to cleave the substrates than that of CtpB, but also that they work together as signal amplifiers to activate downstream proteins in the RIP pathway.

## Introduction

Regulated intramembrane proteolysis was recently identified as a novel cell signaling strategy employed by an increasing number of membrane proteins, and is involved in a number of biological responses such as cellular differentiation (Brown et al., 2000; Lal and Caplan, 2011). RIP not only occurs in animal cells but is also observed in the sporulation of *Bacillus subtilis* (*B. subtilis*), which is a well-studied model for cellular differentiation.

During the process of sporulation in *B. subtilis*, a polar septum partitions the developing cell into two asymmetrically compartments. The larger compartment is known as the mother cell, and the smaller one is referred to as the forespore. The forespore is engulfed by the mother cell, to create a protoplast within a cell (Gomez and Cutting, 1996; Pan et al., 2003; Ganesan et al., 2010). These two cell types differentiate further by activating different σ subunits of RNA polymerase (Delano, 2002; Doerks et al., 2002). The final steps of spore formation are controlled by a sigma factor σ^K^, which is synthesized in the mother cell as an inactive and membrane-associated precursor protein (pro-σ^K^). The pro-σ^K^ is activated by a RIP process (Campo and Rudner, 2006; Mastny et al., 2013; Konovalova et al., 2014) controlled by the processing enzyme SpoIVFB, a membrane-embedded metalloprotease held inactive by two membrane proteins SpoIVFA and BofA. Inhibition of SpoIVFB is relieved by the degradation of negative regulator SpoIVFA (Hoa et al., 2001; Dong and Cutting, 2004), leading to σ^K^ activation and the completion the sporulation program (Resnekov and Losick, 1998; Resnekov, 1999; Kroos et al., 2002).

In *B. subtilis*, activation of this spore RIP pathway is induced by the concerted activity of signaling proteases SpoIVB and CtpB. Both SpoIVB and CtpB belong to the widespread family of PDZ-proteases that combine a catalytic serine protease with a regulatory PDZ domain (Patterson et al., 2005). Crystal structures of CtpB at distinct functional states were reported and showed that CtpB constitutes a ring-like protein scaffold penetrated by two narrow tunnels, and the PDZ domains controls access to the proteolytic sites sequestered within two tunnels (Mastny et al., 2013). The largest subfamily of PDZ-protease is HtrA proteases with DegS as a representative member of the family (Wilken et al., 2004; Israeli et al., 2019). The crystal structure of DegS showed that the protease domains pack together to form a trimer with the PDZ domains located on the periphery locking the protease in inactive form. SpoIVB has been shown to be a new family of trypsin-like serine proteases belonging to clan PA (S55) (Wakeley et al., 2000). However, there is no report regarding the structure of SpoIVB to the best of our knowledge. Structure of SpoIVB has broad significance not only to understand how the PDZ domain regulates the protease activity, but also to the sporulation mechanism.

Here, we report the expression of the recombinant SpoIVB in inactive state (SpoIVB^S378A^) which was fused with an MBP-tag. Combining biochemical and SAXS structural analysis of SpoIVB^S378A^, we found that SpoIVB^S378A^ is a monomer in solution, but not a multimer as in the cases of CtpB and DegS. Comparisons of the PDZ and protease domains to previously reported structures show that the PDZ domain of SpolVB^S378A^ is almost identical to the typical and stable PDZ conformation. However, the protease domain does not form stable active sites and an oxyanion hole. In addition, the combinational pattern of PDZ-protease in SpoIVB is similar to HtrA proteases rather than CtpB. We propose that SpoIVB may adopt a classical cleavage mechanism of trypsin-like serine proteases which is a more directly and simple way to cleave the substrates than CtpB. This model provides new structural information for RIP mechanism.

## Materials and Methods

Benzamidine (CAS number: 206752-36-5) and *p*-aminobenzimidamide (CAS number: 2498-50-2) were purchased from Sigma–Aldrich (St. Louis, MO, United States).

### Construction of SpoIVB Expression Vector

The cDNA encoding truncated SpoIVB (residues Thr75-Ser426, the numbering is according to UniProt entry P17896) was amplified from the full length SpoIVB cDNA [*B. subtilis (strain 168)*, GenBank accession No. AL009126, its cDNA sequence is shown in Supplementary Materials and methods by polymerase chain reaction (PCR) using the primers listed in Supplementary Table S1]. The amplified PCR product was isolated and inserted into the modified expression vector pET22b(+), which has 6 × His-tag and a mMBP fusion tag, a special variant of MBP optimized to improve the possibility of crystallization, between the *Not I* and *Asc I* restriction sites. The construct was transformed into a competent *Escherichia coli* strain DH5α and was then screened on the LB agar plate (0.5% yeast extract, 1% tryptone, 1% NaCl, 2% agar) containing 100 μg/ml Amp to select positive colonies. In order to obtain a stable SpoIVB, we carried out the Site Directed Mutagenesis to mutate Ser378 to Ala (SpoIVB^S378A^). After colony PCR, the recombinant SpoIVB expression vector was verified by sequencing.

### Expression and Purification of SpoIVB^S378A^

In this study, the fusion protein was synthesized in *E. coli* BL21(DE3)pLysS. Expression of SpoIVB^S378A^ was induced by the addition of 0.5 mM IPTG for 16 h at 16°C. Cells were washed by the buffer A (20 mM Tris–HCl, 500 mM NaCl, 5% glycerol pH 8.0) and disrupted by high pressure crushing. After removing debris by centrifugation, cell lysates were purified by passing through IMAC Sepharose^TM^ 6 Fast Flow column (GE Healthcare) equilibrated in buffer A. The column was washed with the buffer A plus 20 mM and 50 mM imidazole and eluted with 300 mM imidazole. Eluted protein was then loaded on Superdex 200 Increase 10/300 GL column (GE Healthcare) previously equilibrated in the buffer A (20 mM Tris–HCl, 500 mM NaCl, 5% glycerol pH 8.0). Flow-through fractions that include SpoIVB^S378A^ were collected and then dialyzed in buffer B (20 mM Tris–HCl, 150 mM NaCl, pH 8.0). Then the purity and concentration of SpoIVB^S378A^ were detected by SDS-PAGE and DS-11 Spectrophotometer (DeNOVIX).

### Dynamic Light Scattering (DLS)

Dynamic light scattering measurements were carried out on a Nano ZS ZEN 3600 (Malvern Instruments, Malvern, United Kingdom) equipped with a 2 ml micro-sampling cell at 25°C. SpoIVB^S378A^ was diluted in a buffer of 20 mM Tris–HCl, 150 mM NaCl, 5% glycerol pH 8.0 to a protein concentration of 0.1, 0.25, and 0.5 mg/ml. All agents were filtered through a 0.22 μM Millipore filter membrane to remove any dust particles before DLS measurement. The cuvette was then inserted into the unit and left to equilibrate for 2 min at 25°C before the measurement. The data were analyzed using the Dynamics software package version 5.

### Small Angle X-Ray Scattering (SAXS) and Structural Modeling of SpoIVB^S378A^

Small angle X-ray scattering measurements of SpoIVB^S378A^ were carried out on the BL19U2 beamline, National Facility for Protein Science Shanghai (NFPSS). All data sets were measured with the exposure time 1 s at 283 K and at a wavelength of λ = 1.0000 Å. Three different concentrations of the protein, 1, 2.5, and 5 mg/ml were used for the measurements. Data for buffers were collected between every two protein samples. The scattering data were then scaled and the average values for the buffers before and after the sample measurements were subtracted. Multiple curves with different concentrations and different exposure times were scaled and merged to generate an ideal average scattering curve. The qualities of the scattering curves were analyzed using the program PRIMUS to ensure that there was no obvious aggregation and radiation damage before further analysis (Konarev et al., 2003). The initial *R*_g_ values were calculated from the Guinier plot, only data from low *q* values were used for the calculation. The P(r) distribution function was calculated with the program GNOM (Svergun, 1992). The molecular weight was estimated directly from the SAXSMoW server^1^ using the P(r) distribution function (Fischer et al., 2010). Data were also processed and analyzed using SCATTER (Rambo and Tainer, 2013).

The low-resolution global shape of the protein in solution was modeled by the program DAMMIF (Franke and Svergun, 2009) in the asymmetric unit and P1 symmetry using both the original scattering curve and the calculated P(r) distribution curve. Twenty individual calculations were performed. Subsequently, continuous and meaningful shapes were picked up and averaged by the program DAMAVER (Volkov and Svergun, 2003). The starting model of mMBP was extracted from the published crystal structure of MBP (PDB code: 1ANF) (Quiocho et al., 1997) and updated its amino acid sequence. The rest part of SpoIVB^S378A^, including the PDZ and protease domains, were modeled by the SWISS-MODEL server^2^ based on their sequence homologies (Bienert et al., 2017), which used previous published PDZ (PDB codes: 1QAV) and protease (PDB codes: 1DLE) domains as the templates (Hillier et al., 1999; Jing et al., 2000). And then mMBP, PDZ, and protease domains were superimposed into the SAXS envelope of SpoIVB^S378A^ and adjusted them manually by using the program Chimera (Pettersen et al., 2004), respectively. Hexamer His-tag at the N-terminal of SpoIVB^S378A^ was built in the model by COOT (Emsley et al., 2010).

### Molecular Dynamic Simulation

Molecular dynamic simulations were performed on modeling of SpoIVB^S378A^. No atom on the protein was within 13 Å from any side of the box. Sodium ions were added to counterbalance the charge of the protein. All systems were underwent MD simulations with AMBER ff99SB-ILDN force field (Walker et al., 2002; Craft and Legge, 2005; Lindorfflarsen et al., 2010) using the Amber 16 code (Case et al., 2010). The TIP3P model (Degryse, 2008) was used for the water molecules. PME (Darden et al., 1993) method was used to treat electrostatic interactions, and all covalent bonds containing hydrogen atoms were constrained using SHAKE algorithm. The cutoff distance applied for the non-bond interactions was taken at 10 Å. Each system underwent 25,000 steps of steepest-descent energy minimization, followed by 25,000 steps of conjugate-gradient minimization without restraints. The system was then gradually heated from 0 up to 298 K in 25,000 steps. After that, 100-ns-long MD simulations were carried out in the NPT ensemble for each system. Temperature and pressure controls were achieved by Nosé-Hoover thermostat (Liu et al., 2017) and Berendsen barostat (Berendsen et al., 1984) with a frequency of 2.0 ps, respectively. FoXS was used for computing a SAXS profile of the free energy optimized SpoIVB^S378A^ and for matching of the computed and experimental profiles (Schneidman-Duhovny et al., 2010).

### Microscale Thermophoresis (MST) Analysis of SpoIVB^S378A^ Binding to Benzamidine

SpoIVB^S378A^ was labeled using the Protein Labeling Kit RED-NHS (NanoTemper Technologies). The labeling reaction was performed according to the manufacturer’s instructions in the supplied labeling buffer applying a concentration of 10 μM protein (molar dye: protein ratio ≈ 2:1) at 4°C temperature for 2 h in the dark. Unreacted dye was removed with the supplied dye removal column equilibrated with PBS contained 0.05% Tween-20. The degree of labeling was determined using UV/VIS spectrophotometry at 650 and 280 nm. A degree of labeling of 0.8 was typically achieved. The labeled protein SpoIVB^S378A^ was adjusted to 1 μM with PBS buffer supplemented with 0.05% Tween-20. The ligand benzamidine was dissolved in PBS buffer supplemented with 0.05% Tween 20. For the measurement, each ligand dilution was mixed with one volume of labeled protein SpoIVB^S378A^, which led to a final concentration of SpoIVB^S378A^ of 500 nM and final ligand concentrations ranging from 15 μM to 500 mM. After 15 min incubation followed by centrifugation at 10,000 × *g* for 5 min, the samples were loaded into Monolith NT.115 [Premium] Capillaries (NanoTemper Technologies). MST was measured using a Monolith NT.115 [NT.115Pico/NT.LabelFree] instrument (NanoTemper Technologies) at an ambient temperature of 25°C. Instrument parameters were adjusted to 80% LED power and medium MST power. Data were analyzed using NanoTemper Analysis software v. 2.4 and plotted using the GraphPad Prism v.6.01 software from GraphPad Software, Inc.

### Detection of the Binding Between SpoIVB^S378A^ and *p*-Aminobenzamidine (PAB) by Fluorescence Spectra

*p*-Aminobenzamidine can be used as a typical fluorescence probe to assay the accessibility of active site of serine proteases by determining the equilibrium binding constants (*K*_D_). Fluorescence emission spectra were recorded at 25°C on a fluorescence spectrometer (Cary Eclipse, Varian, United States) in a 2 mm × 10 mm semi-micro quartz cuvette and in PBS buffer. Excitation was at 335 nm, and the emission was scanned from 350 to 400 nm using excitation and emission band passes of 5 and 10 nm, respectively. Equilibrium binding reactions were performed over a range of PAB concentrations (100–1000 μM) with 1 μM SpoIVB^S378A^ in PBS buffer. For positive control, binding reactions of PAB with 1 μM serine protease domain of urokinase plasminogen activator (uPA-SPD) were also performed, respectively. Emission spectra were collected using an integration of 1–2 s over a 1.0 nm step resolution. Data were analyzed and plotted using the GraphPad Prism v.6.01 software from GraphPad Software, Inc.

## Results and Discussion

### Expression and Purification of SpoIVB^S378A^

**FIGURE 1:**
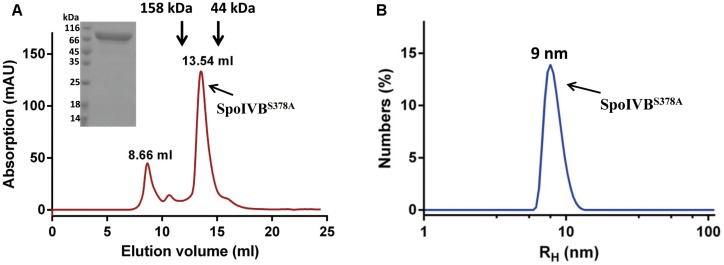
Recombinant SpoIVB^S378A^ is monodispersed and exists as monomer in the solution. **(A)** SpoIVB^S378A^ was purified to high purity by a gel filtration column (Superdex increase 200 10/300 GL). The retention volume of 13.54 ml corresponds to the monomeric form of SpoIVB^S378A^. Arrows indicate the elution positions of protein molecular mass standards that were run separately on the same column. Retention volumes are shown for the molecular weight standards and 15% SDS-PAGE analysis of the elution fraction corresponding to the peak. **(B)** Dynamic light scattering data for SpoIVB^S378A^. Count rate = 173.6 kcps and polydispersity index (PdI) = 0.204. The hydrodynamic molecular radius of SpoIVB^S378A^ is about 9 nm.

Since SpoIVB zymogen can be auto-activated, its expression in *E. coli* is toxic to the host, resulting in low yield of the recombinant protein as previously described (Dong and Cutting, 2004; Campo and Rudner, 2006). In our hands, we found that the expression level of the SpoIVB (Thr75-Ser426) fused to the modified MBP (mMBP) as an N-terminal tag was quite low. To address this problem, we mutated the catalytic residue Ser378 to Ala (SpoIVB^S378A^), which led to an increased expression level (30 mg/l). The recombinant SpoIVB^S378A^ was captured from the cell lysates in one step by a Ni-NTA agarose column and further purified by size exclusion chromatography (SEC). This gave a peak with a retention volume of about 13.54 ml, which is between one standard protein (158 kDa, 12.77 ml) and the other standard protein (44 kDa, 15.30 ml). The pooled protein was detected as a predominant band of ∼83 kDa by SDS-PAGE, which is consistent with the predicted molecular weight of the truncated SpoIVB infused the mMBP (80.5 kDa, Figure 1A).

### SpoIVB^S378A^ Is Monodispersed and Exists as Monomer in the Solution

The chromatography profile of SpoIVB^S378A^ showed that protein existed predominantly as a monomer. Next, we used DLS to analyze the size of SpoIVB^S378A^ at three concentrations (0.1, 0.25, and 0.5 mg/ml). The size of distribution of SpoIVB^S378A^ showed a single species with the hydrodynamic radii (R_H_) at about 9 nm (Figure 1B), corresponding to a molecular weight of around 80 kDa based on a spherical model. These results demonstrated that SpoIVB^S378A^ was monodispersed and did not form multimer.

### Ligand Binding Conformation of SpoIVB^S378A^

**FIGURE 2:**
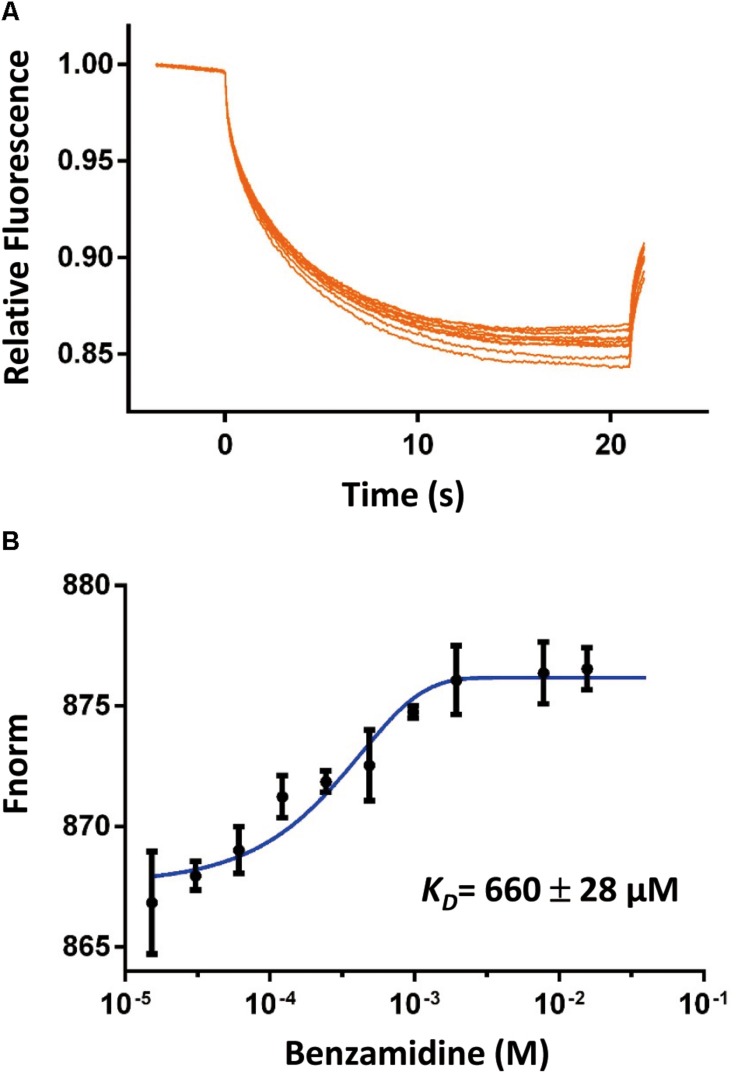
The binding affinity of benzamidine toward SpoIVB^S378A^ was quantified by MicroScale Thermophoresis (MST). **(A)** Comparison of MST traces of titrations of benzamidine against 500 nM SpoIVB^S378A^. **(B)** The *K*_D_ of this interaction was determined to be 660 ± 28 μM (*n* = 2).

To address if the SpoIVB^S378A^ active site was accessible, we determined its binding affinities to two classical serine proteases inhibitors by different methods to probe the accessibility of the active site. The first inhibitor is benzamidine, which is a reversible competitive inhibitor of serine proteases and has a binding affinity range in 5–20 μM for the active serine proteases (Tanizawa et al., 1971; Hedstrom et al., 1996). We used the MST method to measure the binding affinity of benzamidine for SpoIVB^S378A^ and found that benzamidine bound to SpoIVB^S378A^ with a rather weak affinity (*K*_D_ = 660 ± 28 μM) (Figure 2). The second inhibitor we used is PAB which is also a fluorescent probe for the active site of serine proteases, with excitation and emission maxima at 293 and 376 nm, respectively. Binding to serine proteases typically results in a blue shift of the emission peak to 362–368 nm, and 50–230 fold fluorescence enhancement (Evans et al., 1982). Mostly, the *K*_D_ of PAB to serine proteases is about 10–100 μM (Evans et al., 1982). For SpoIVB^S378A^ titrated with increasing concentrations of PAB, we found no fluorescence enhancement. However, in the positive control when the active uPA-SPD titrated with increasing concentrations of PAB, the fluorescence enhancement was found, indicating that the active site of SpoIVB^S378A^ was not properly formed (Supplementary Figure S2).

### Small Angle X-Ray Scattering (SAXS) Analyses of SpoIVB^S378A^

**FIGURE 3:**
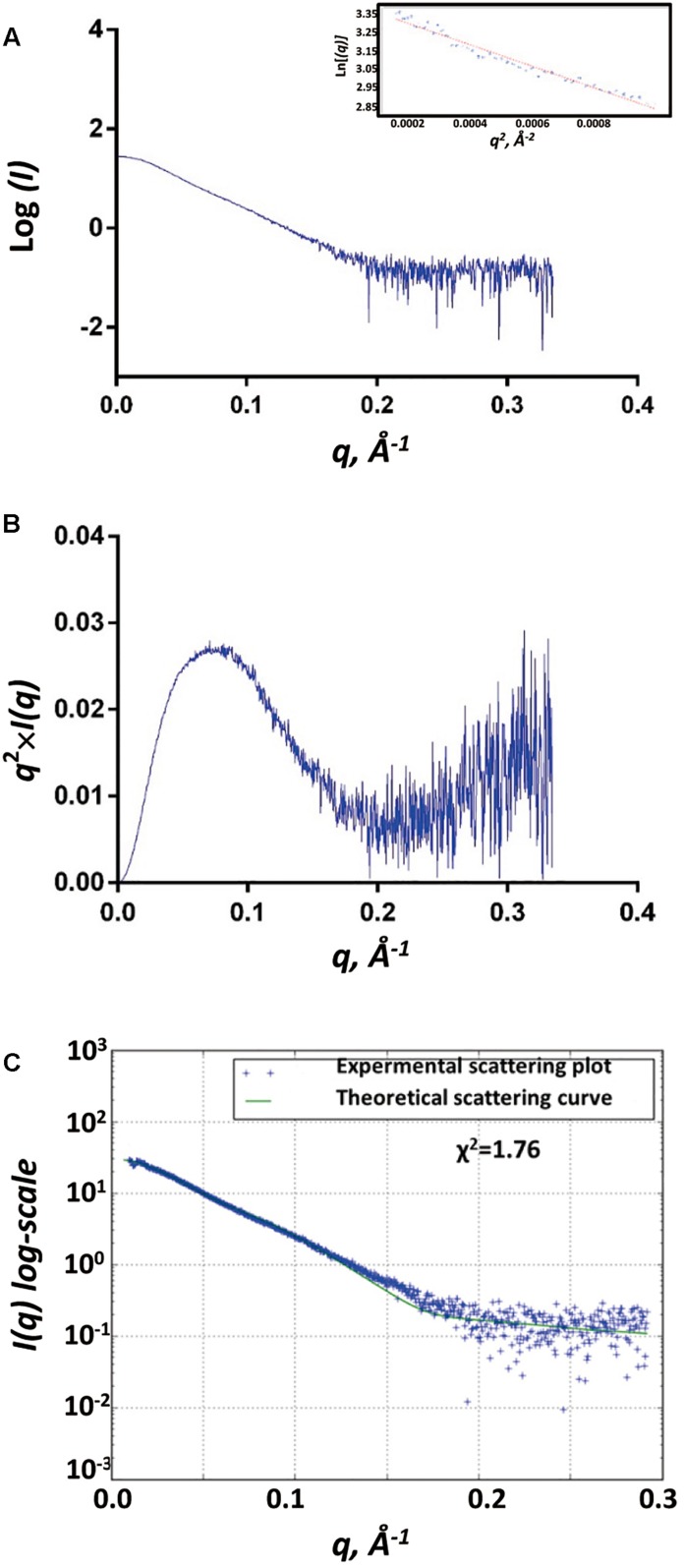
SAXS analyses of SpoIVB^S378A^. **(A)** Experimental SAXS scatter plot of SpoIVB^S378A^ in solution. The Guinier region and the corresponding linear fitting are shown in the inset. **(B)** Kratky plot calculated from the experimental data. **(C)** Superposition of experimental and theoretical scatter curves using FoXS. The experimental scattering plot of SpoIVB^S378A^ (blue dots) agrees well with the theoretical scattering curve (green line) obtained from the models (χ^2^ = 1.76).

We used the biological SAXS (BioSAXS) experiment to study the structure of SpoIVB^S378A^ in solution at series of concentrations (1, 2.5, and 5 mg/ml). We did not find unspecific aggregation of SpoIVB^S378A^ in the highest concentration (5 mg/ml), and thus used this concentration for BioSAXS shape reconstruction and modeling (Figure 3A). Kratky analysis was used to evaluate the degree of protein folding, which showed a peak at low *q* values and return to increase at high *q* values, indicating SpoIVB^S378A^ is folded but somewhat flexible in solution (Figure 3B).

The gyration radii of SpoIVB^S378A^ (*R*_g_) was measured to be 37.6 Å. The molecular weight of 77.6 kDa for SpoIVB^S378A^ was obtained from SAXSMoW using an integration range restricted to *qR*_g_ < 1.3 and *q*_max_ limited to *I*(0)*/I*(*q*_m_) = 10^2.25^ (Table 1). This value is close to the obtained molecular weight (80.5 kDa) based on the amino acid sequence and reflects that SpoIVB^S378A^ is a monomer, consistent with the results of SEC and DLS measurements.

**TABLE 1:** The collection and molecular weight parameters of BioSAXS data of SpoIVB^S378A^.

**Sample**	**Exposure time (s)**	**Wavelength (Å)**	***R_g_* (Å)**	**q × *R_g_***	**Molecular weight from SAXSMoW (kDa)**	**Molecular weight from amino acid sequence (kDa)**
SpoIVB^S378A^	1	1.0000	37.641	1.289	77.6	80.5

The recombinant SpoIVB^S378A^ construct contains a hexamer His and mMBP tags at the N-terminal. mMBP tag is not only beneficial to purify the recombinant protein, but also conducive to determine the BioSAXS structure and evaluate the reliability of the model. The BioSAXS molecular envelope of SpoIVB^S378A^ showed appropriate space to fit the mMBP. Once mMBP was positioned inside the molecular envelope, the position of the PDZ and protease domains can be placed easily. Because the structure of SpoIVB is still unknown, we use the SWISS-MODEL server to reconstruct three-dimensional structures for the PDZ and protease domain, respectively. This model was then fitted into the molecular envelope of SAXS using the program Chimera and was energy-minimized by MDs (Supplementary Figure S1) to obtain the final solution structure of SpoIVB^S378A^. The theoretical scattering pattern computed from the modeled structure of SpoIVB^S378A^ fits very well to the experimental data (χ^2^ = 1.76) (Figure 3C).

**FIGURE 4:**
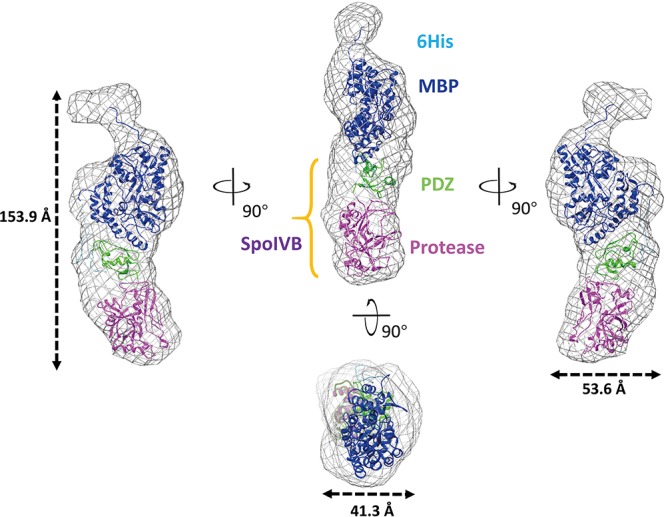
Overall structure of SpoIVB^S378A^ as determined by BioSAXS. Superimposition of the BioSAXS envelope (shown as a gray mesh) onto SpoIVB^S378A^ is represented as ribbons. SpoIVB^S378A^ includes the 6 × His (colored as light blue) and mMBP (colored as blue) tags at the N-terminal, PDZ domain (colored as green) in the middle, and protease domain (colored as magenta) at the C-terminal.

In the overall structure, SpoIVB^S378A^ molecular envelope has a cylinder shape with a dimension of approximately 153.9 Å × 53.6 Å × 41.3 Å (Figure 4). The mMBP tag did not interact with PDZ domain and should not affect the conformation of other parts of recombinant protein, which was also the case as reported in previous studies (Waugh, 2016). The active site mutation has localized effect and is limited to the active site and will most likely not affect such domain orientation. There is only one interaction surface between two loops that is in PDZ and protease domain, respectively. The first loop locates on between the αB helix and βD strand of the PDZ domain, the other one locates on Gly252-Thr258 segment of protease domain (Supplementary Figure S3). Comparing with other reported PDZ domain structures, we found the PDZ domain in SpoIVB also adopts a conserved fold, which usually has five β strands as well as a short and long α helix (Morais Cabral et al., 1996). In contrast to the PDZ domain, the protease domain of SpoIVB^S378A^ adopts a non-canonical inactive protease conformation, which is a zymogen-like conformation with no oxyanion hole and a proper catalytic triad in SpoIVB^S378A^ (Supplementary Figure S4A). Hence, we propose that SpoIVB activation is a disorder–order transition that exits in other previously reported classical trypsin-like proteases activation. Such as in the inactive trypsinogen, the activation motif is highly flexible, whereas in the active trypsin protease it occupies a well-defined position poised for catalysis (Wilken et al., 2004). The other example can be found in the active uPA, the activation motif and the S1 pocket is well-defined position poised for catalysis. But when uPA is inactive, the activation motif is highly flexible and the S1 pocket is disordered (Jiang et al., 2013).

Dong and Cutting (2004) proposed that there are five steps to process the activation of SpoIVFB, including *trans* and *cis* cleavages. First, the PDZ domain of SpoIVB binds to its N-terminus to maintain in its zymogen form. Following secretion across the spore membrane, binding of one SpolVB molecule in *trans* to another SpolVB C-terminus occurs. This facilitates the first cleavage event of SpoIVB near the N-terminus, releasing it from the inner forespore membrane. Then at least two further *cis* cleavage events occur at specific sites near the N-terminus after which the PDZ domain targets SpoIVB to the BofA-SpoIVFA-SpoIVFB complex in the outer forespore membrane, in turn, activating the SpoIVFB by disruption of the complex.

During the second step of activating SpolVFB, it has been proposed that SpolVB must first bind to a C-terminal recognition Thr393-His-Val395 motif (TXV motif). This is identical to the classical PDZ recognition motif (Songyang et al., 1997; Beebe et al., 2000) and ensures trans cleave normally proceeds. However, we found that the TXV motif of SpoIVB^S378A^ locates at the opposite direction to the PDZ domain. This indicates that the PDZ domain does not make any intramolecular interactions with protease via the TXV motif (Supplementary Figure S4B). This may cause it to fail to complete the second step *trans* cleavage and the following steps.

### Comparisons of SpoIVB With Other PDZ-Protease Family Members

Regulated intramembrane proteolysis is a method for transducing signals between cellular compartments. In the process of sporulation, two signaling serine proteases, SpoIVB and CtpB, trigger pro-σ^K^ processing by cleaving the extracellular domain of SpoIVFA at multiple sites. Following this, inhibition of SpolVFB is relieved. SpoIVB cooperates with CtpB ensuring the precise temporal control of transferring an activating signal from forespore to mother cell. However, it is still unclear why the two distinct proteases must be involved and how they cooperate with each other in same process of cleaving the regulator protein SpoIVFA.

First, we compared the structures of PDZ and protease domain in SpoIVB^S378A^ and CtpB at the resting state (PDB code: 4C2E; Mastny et al., 2013). We found that the PDZ-protease patterns are obvious different to each other (RMSD = 23.1 Å). As described above, the PDZ and protease domains do not have any interface in SpoIVB^S378A^ except for the two loops region. However, in the resting state of CtpB, PDZ domain gated the protease tunnel leading to CtpB unable to hydrolyze SpoIVFA. Comparing SpoIVFA cleavage sites targeted by SpoIVB (four cleavage sites) and CtpB (one cleavage site), we proposed that SpoIVB might adopt a different and higher efficient regulation way to trim the SpoIVFA substrate than CtpB.

**FIGURE 5:**
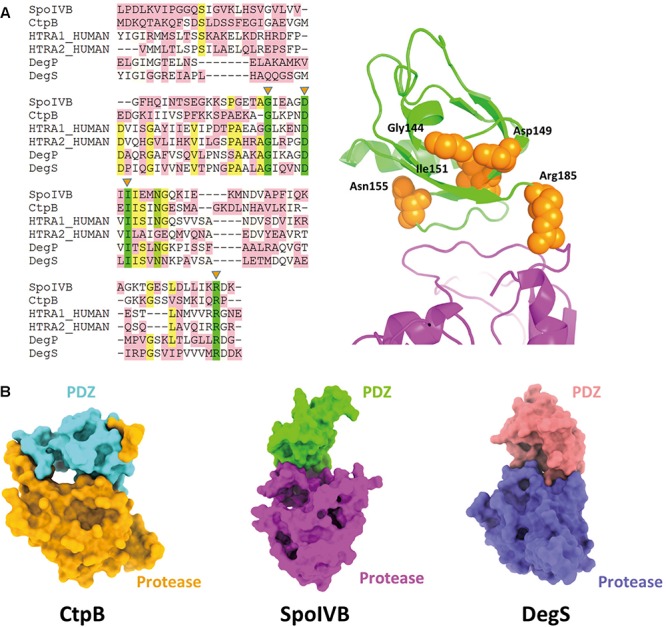
Comparisons of SpoIVB with other PDZ-protease family members. **(A)** Sequence alignment of SpoIVB, CtpB, HtrA1, HtrA2, DegP, and DegS. Left side: There are five high conservative residues include Gly144, Asp149, Ile151, Asn155, and Arg185 labeled by orange triangles. Right side: These five conservative residues (shown in orange balls) locate on one face of the PDZ domain that points to the protease domain. **(B)** The pattern of PDZ-protease in SpoIVB is different with that in CtpB; SpoIVB adopts a similar pattern with DegS.

Except SpoIVB and CtpB, there are the widespread family of PDZ-proteases. The HtrA proteases constitute the largest subfamily of PDZ-proteases including HtrA1, HtrA2, DegP, and DegS (Clausen et al., 2011; Zhu et al., 2017). Although their protease domains belong to different classes, they share a homologous PDZ domain. There are five high conservative residues in the PDZ domains including Gly144, Asp149, Ile151, Asn155, and Are185 (based on SpoIVB sequence numbering). More strikingly, these residues locate on one face of the PDZ domain, which points to the protease domain and may play a role in regulate the substrate access to the active site of SpoIVB (Figure 5A).

It is a surprise that SpoIVB^S378A^ was monodispersed and did not form a multimer. This is different from the other PDZ-proteases, such as CtpB and DegS. The truncated DegS was trimeric in solution while CtpB was a dimer. Why SpoIVB^S378A^ is monomer? This may be because the mutation of the active site Ser378 to Ala made protease domain unstable and the S1 pocket more flexible. Structurally, the PDZ domain cannot make any intramolecular interactions with protease domain via the TXV motif in the solution structure of SpoIVB^S378A^. This may lead to SpoIVB no being to form *trans*-dimer and exists as a monomer.

Zeth (2004) and Clausen et al. (2011) reported that the crystal structures of DegS and HtrA2 protease domains in an inhibited state, respectively (Wilken et al., 2004; Zeth, 2004; de Regt et al., 2015). These structures indicate that the proteases is present in a non-functional state without an oxyanion hole and a proper catalytic triad and unlike CtpB, in inactive DegS, the PDZ domain did not block the access to the active site. This is similar to the combinational pattern of PDZ-protease in SpoIVB (Figure 5B). Hence, we propose that the activation regulation of SpoIVB and HtrA proteases are in a similar way.

## Data Availability

SAXS data were deposited at the Small Angle Scattering Biological Data Bank (SASBDB) (Valentini et al., 2015) under codes SASDFJ3.

## Author Contributions

LJ and MH: conceptualization. XX, NG, GX, and DX: experiments. CY, JH, and LJ: data analysis. CY, MH, and LJ: funding acquisition. LJ and MH: project administration. MH: resources. GX and DX: software. MH and LJ: supervision. MH and LJ: validation. LJ: visualization. XX, DX, JL, and LJ: writing – original draft. XX, JH, MH, and LJ: writing – review and editing.

## Conflict of Interest Statement

The authors declare that the research was conducted in the absence of any commercial or financial relationships that could be construed as a potential conflict of interest.

## Footnotes

1 http://saxs.ifsc.usp.br/

2 https://www.swissmodel.expasy.org/

## Abbreviations

CtpB: C-terminal processing proteases B
DLS: dynamic light scattering
FoXS: fast X-ray scattering
HtrAs: high temperature requirement A proteases
IPTG: isopropyl 1-thio-*β*-D-galactopyranoside
MBP: maltose binding protein
MD: molecular dynamic
MST: microscale thermophoresis
PAB: *p*-aminobenzamidine
PdI: polydispersity index
PME: particle mesh Ewald
RIP: regulated intramembrane proteolysis
SAXS: small angle X-ray scattering
SpoIVB: sporulation factor IV B protease
SpoIVFA: stage IV sporulation protein FA
SpoIVFB: stage IV sporulation protein FB.
