# Supplementary material for: Solution Structure of SpoIVB Reveals Mechanism of PDZ Domain-Regulated Protease Activity
Source: Front Microbiol. 2019 Jun 12;10:1232. doi: 10.3389/fmicb.2019.01232 (PMC6581720; doi:10.3389/fmicb.2019.01232)
Supplement: Supplementary file 1 [file Data_Sheet_1.docx]

Supporting information for

**Solution Structure of SpoIVB Reveals Mechanism of PDZ Domain-regulate Protease Activity**

Xie Xie^1^, Nanna Guo^1^, Guangpu Xue^1^, Daoqin Xie^1^, Cai Yuan^2^, Joshua Harrison ^3^, Jinyu Li^1^, Longguang Jiang^1, *^, Mingdong Huang^1, *^

^1^ College of Chemistry, Fuzhou University, Fuzhou 350116, China

^2^ College of Biological Science and Engineering, Fuzhou University, Fuzhou 350116, China

^3^ Beth Israel Deaconess Medical Center, Harvard Medical School, Boston 02115, USA

^*^ Co-corresponding authors: Longguang Jiang (jianglg@fzu.edu.cn); Mingdong Huang (HMD_lab@fzu.edu.cn)

**MATERIALS AND METHODS**

**The DNA encoding the full length SpoIVB (GenBank accession No. AL009126):**

ATGCCCGATAACATCAGAAAAGCAGTAGGTTTAATTCTCCTTGTTTCGTTATTAAGTGTAGGTTTATGCAAACCGCTAAAAGAATATTTACTGATTCCAACGCAAATGAGAGTATTTGAAACCCAAACACAAGCGATTGAAACGAGTTTATCGGTAAACGCTCAGACATCAGAATCCTCAGAAGCGTTTACAGTAAAGAAAGATCCGCATGAAATCAAGGTGACGGGCAAAAAATCAGGTGAGTCAGAATTGGTATATGATCTTGCCGGATTTCCAATTAAAAAAACAAAAGTGCATGTTCTTCCTGATTTAAAAGTTATACCTGGCGGACAATCAATCGGTGTAAAACTTCATTCCGTCGGTGTTCTTGTCGTCGGATTTCATCAAATCAATACAAGTGAAGGCAAAAAATCTCCGGGAGAAACGGCAGGAATTGAAGCGGGCGACATCATTATTGAGATGAATGGACAGAAAATTGAAAAAATGAATGATGTAGCCCCATTTATTCAAAAGGCTGGGAAAACTGGTGAATCTTTAGACTTACTGATCAAACGTGATAAACAGAAAATCAAAACGAAGCTGATCCCAGAAAAGGATGAAGGAGAAGGCAAATACAGAATCGGGTTATATATCAGAGATTCTGCTGCTGGCATCGGCACTATGACCTTTTATGAACCGAAAACAAAAAAATACGGAGCACTTGGCCACGTGATTTCCGATATGGACACAAAGAAACCAATTGTAGTGGAGAATGGAGAAATCGTTAAATCCACTGTAACATCAATTGAAAAAGGGACAGGCGGTAATCCGGGAGAAAAACTGGCGCGATTTTCCTCAGAACGCAAAACGATCGGGGATATTAACAGAAACAGCCCGTTTGGGATTTTCGGCACACTGCATCAGCCGATTCAAAACAACATATCAGATCAAGCATTGCCGGTTGCGTTTTCTACCGAAGTCAAAAAAGGGCCGGCTGAAATTTTAACGGTTATTGATGATGACAAAGTAGAAAAATTCGATATTGAAATCGTCAGCACAACGCCGCAAAAATTCCCTGCGACAAAAGGAATGGTGTTGAAAATTACCGATCCAAGACTGTTGAAAGAAACAGGAGGCATCGTACAGGGGATGAGCGGAAGCCCGATCATTCAAAATGGAAAAGTGATCGGTGCTGTCACCCATGTATTTGTAAATGACCCGACAAGCGGCTACGGTGTTCATATTGAATGGATGCTGTCAGAAGCAGGAATCGATATTTATGGAAAAGAAAAAGCAAGCTGA

**The DNA encoding mMBP:**

AAAACCGAAGAAGGTAAACTGGTAATCTGGATTAACGGCGATAAAGGCTATAACGGTCTCGCTGAAGTCGGTAAGAAATTCGAGAAAGATACCGGAATTAAAGTCACCGTTGAGCATCCCGATAAACTGGAAGAGAAATTCCCACAGGTTGCGGCAACTGGCGATGGCCCTGACATTATCTTCTGGGCACACGACCGCTTTGGTGGCTACGCTCAATCTGGCCTGTTGGCTGAAATCACCCCGGCCGCAGCGTTCCAGGACAAGCTGTATCCGTTTACCTGGGATGCCGTGCGCTACAACGGCAAGCTGATTGCTTACCCGATCGCTGTTGAAGCGTTGTCCCTGATTTATAACAAGGATCTGCTGCCGAACCCGCCAAAAACCTGGGAAGAGATCCCGGCGCTGGATAAAGAACTGAAAGCGAAAGGTAAGAGCGCGCTGATGTTCAACCTGCAAGAACCGTACTTCACCTGGCCGCTGATTGCTGCTGACGGGGGTTATGCGTTCAAGTATGCAGCCGGAAAGTACGACATTAAAGACGTGGGCGTGGATAACGCTGGCGCGAAAGCGGGTCTGACCTTCCTGGTTGACCTGATTAAAAACAAACACATGAATGCAGACACCGATTACTCCATCGCAGAACATGCCTTTAATCATGGCGAAACAGCGATGACCATCAACGGCCCGTGGGCATGGTCCAACATCGACACCAGCGCAGTGAATTATGGTGTAACGGTGCTGCCGACCTTCAAGGGTCAACCATCCAAACCGTTCGTTGGCGTGCTGAGCGCAGGTATTAACGCCGCCAGTCCGAACAAAGAGCTGGCAAAAGAGTTCCTCGAAAACTACCTGCTGACTGATGAAGGTCTGGAAGCGGTTAATAAAGACAAACCGCTGGGTGCCGTAGCGCTGAAGTCTTACGAGGAAGAGTTGGTGAAAGATCCACGTGTTGCCGCCACAATGGAAAACGCCCAGAAAGGTGAAATCATGCCCAACATCCCGCAGATGTCCGCTTTCTGGTATGCCGTGCGCACTGCGGTGATTAACGCCGCCAGCGGTCGTCAGACTGTCGATGCAGCCCTGGCAGCCGCGCAGACTAAT

**Table S1. Primers for PCR, site-directed mutagenesis in this study.**

| **Primer name** | **Sequence^a^** | **Usage** |
| --- | --- | --- |
| SpoIVB^T75^_F | ATAAGAATGCGGCCGCTACCGGTAAAAAATCTGG | PCR |
| SpoIVB^T75^_R | TTGGCGCGCCTTAACTGGCTTTTTCTTTAC | PCR |
| SpoIVB^S378A^_F | GCAGGGTATGgcaGGTAGCCCGATTATTC | Mutation:  S378A |
| SpoIVB^S378A^_R | ACAATACCACCGGTTTCTTTC | Mutation:  S378A |

^a^ The underlined letters indicate restriction sites, and the lowercase letters indicate mutated sites.

**
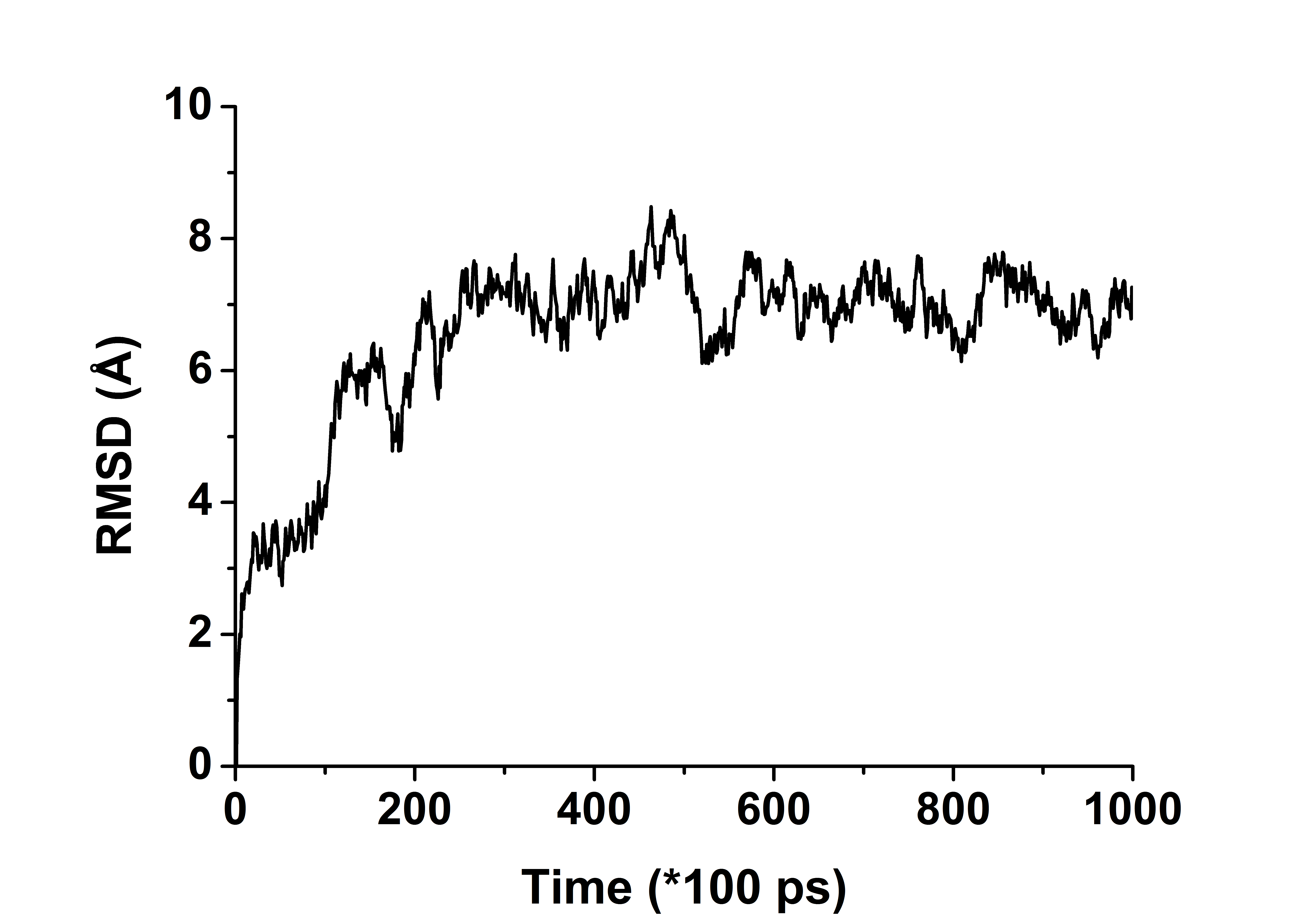
**

**Figure S1. MD free energy optimization of the SAXS model shows the model stabilized at 20ns.** RMS deviations from starting structures were evaluated for backbone C, N, and CA atoms during the trajectory.

**
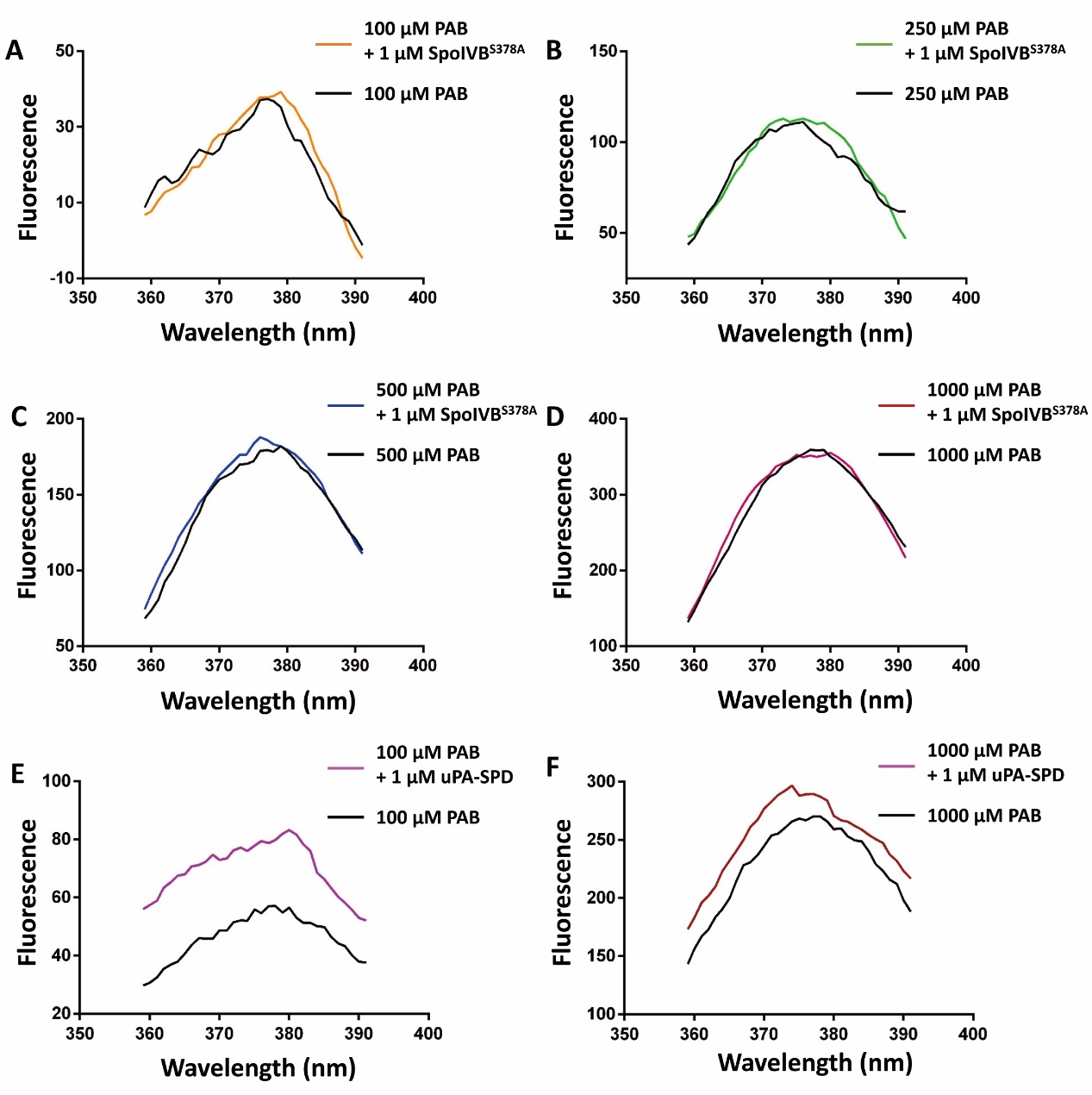
**

**Figure S2. Fluorescence spectra of the titration of SpoIVB^S378A^ and uPA-SPD with different concentrations of PAB.** A: 100 μM PAB with 1 μM SpoIVB^S378A^; B: 250 μM PAB with 1 μM SpoIVB^S378A^; C: 500 μM PAB with 1 μM SpoIVB^S378A^; D: 1000 μM PAB with 1 μM SpoIVB^S378A^; E: 100 μM PAB with 1 μM uPA-SPD; F: 1000 μM PAB with 1 μM uPA-SPD. There are no significate fluorescence enhancement in the titration of SpoIVB^S378A^ with PAB, however, there are significate enhancement in the titration of uPA-SPD with PAB.


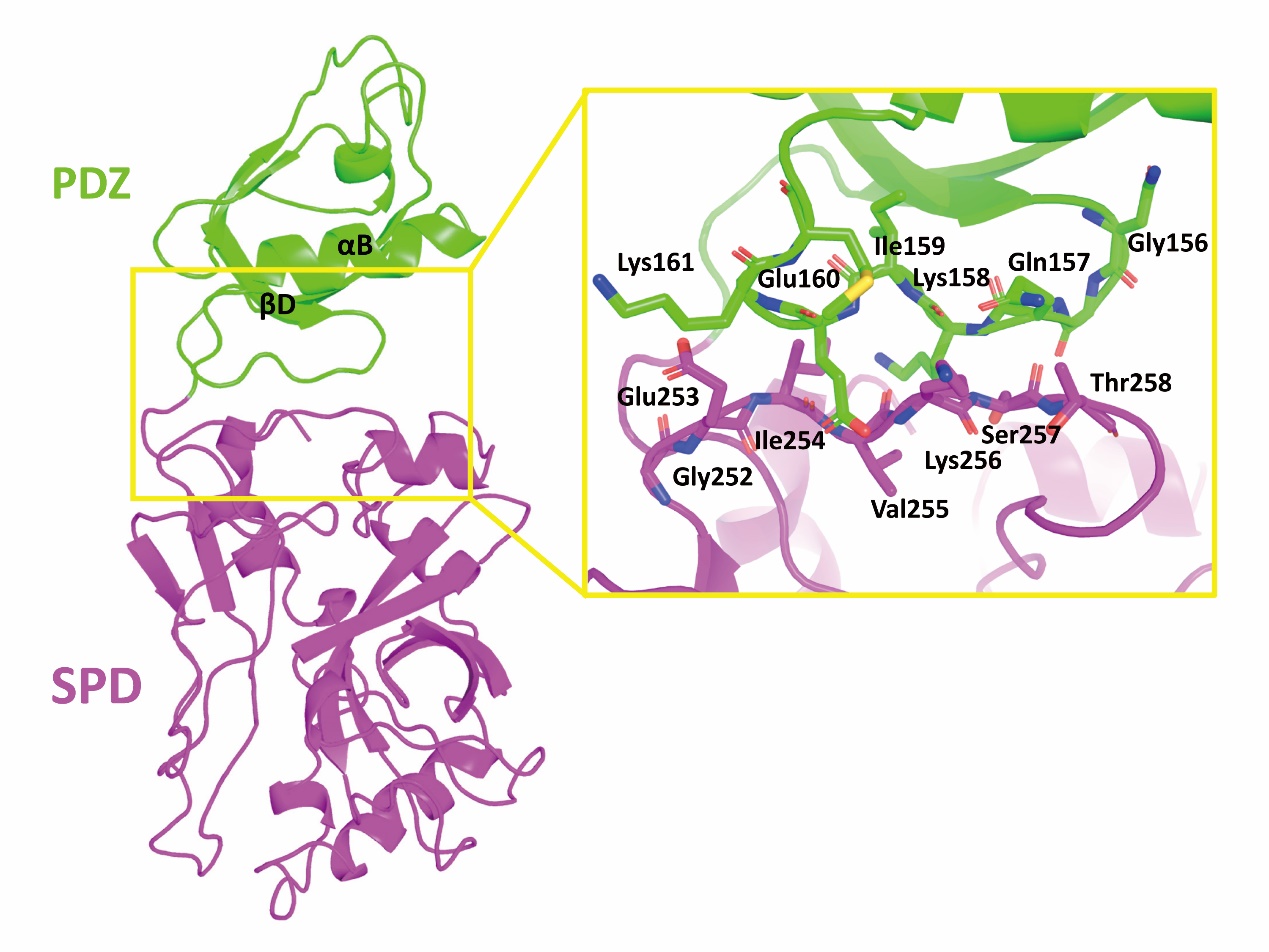


Figure S3. Molecular model of SpoIVB and the interface between PDZ and protease domain.

**
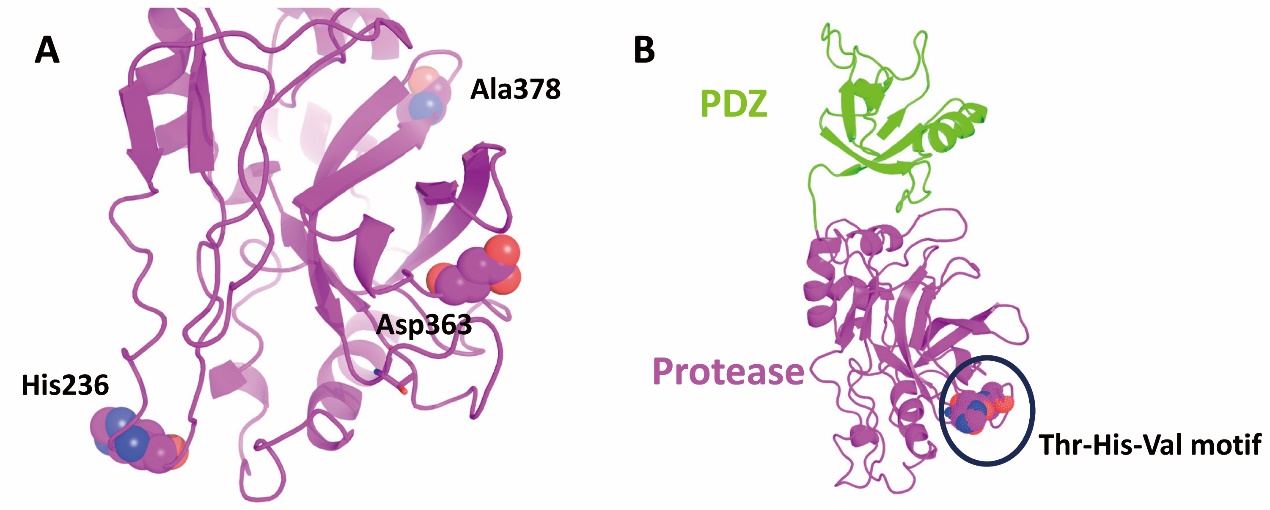
**

Figure S4. A: SpoIVB^S378A^ has no oxyanion hole and proper catalytic triad. B: The PDZ domain does not make any intramolecular interactions with SPD via the TXV motif.
